# Fluorescent polyacrylate derivate from 4-biphenylmethanol with UV-green emission

**DOI:** 10.1080/15685551.2018.1514703

**Published:** 2018-09-22

**Authors:** Ana M. Herrera-González, Martín Caldera-Villalobos, Jesús García-Serrano, Marissa Vargas Ramírez, Azdrubal Lobo Guerrero-Serrano

**Affiliations:** Instituto de Ciencias Básicas e Ingeniería, Universidad Autónoma del Estado de Hidalgo, Mineral de la Reforma, México

**Keywords:** Fluorescence, biphenyl, polyacrylate, blue-light emitter, UV-light emitter, short-wavelenght emitter

## Abstract

In this paper we report the synthesis of a new polyacrylate named poly(1,1ʹ-BP4MA) which is a derivate from 4-biphenylmethanol monomer. Poly(1,1ʹ-BP4MA) was obtained by solution and bulk polymerization techniques to yield polymers with high molecular weight and high solubility. The study of the optical properties showed that poly(1,1ʹ-BP4MA) is a fluorescent material with emission in the UV-green region and it has similar quantum yield to tryptophan.

## 1. Introduction

The short-wavelength radiation from solid state sources it is important for the construction of full colour displays because different phosphors can be excited by UV, violet or blue light to obtain light with higher wavelengths [1]. Further, short-wavelength emitter materials are interesting for construction of high density information storage devices [2].

However, it is difficult to obtain appropriate materials to fabricate OLEDs with efficient emission of UV or blue light. Some properties required for this are wide band gap, high quantum yield, high solubility and capability to form films with controlled morphology. To achieve the wide band gap it is necessary to confine the extent of conjugation imposing constraints in the molecular size or introducing a twist between conjugated rings. This implies the design of non-planar conjugated structures [3]. Despite poly(*p*-phenylenes) are still the most employed organic materials for short-wavelength emitters, they exhibit poor solubility in organic solvents and they are difficult to process [4]. Thus, it is necessary to design novel organic materials with UV-Blue emission.

Highly efficient blue-light organic emitter materials like polymers, oligomers and compounds are typically based in conjugated structures containing fused rings, e.g., naphthalene, anthracene, pyrene and fluorene moieties [5–11]. Also, heterocyclic moieties with fused rings like carbazole, quinoline and dibenzothiophene have been employed to obtain organic blue light emitters [12–20].

However, there is not much report about satisfactory materials for UV and violet light emitters. Some examples of organic materials containing fluorene groups with emission around 400 nm have been reported recently [21]. An alternative for the obtaining of organic short-wavelength emitters are materials with biphenyl and terphenyl groups [22–24]. These groups contain conjugated aromatic rings connected by single C-C bonds with free rotation, which increase the value of band gap [25,26].

Previous works have reported the study of the spacial order in polymers containing biphenyl pendant groups. However, these polymers do not have any application until now [27,28].

Taking account this background, in this work we report the synthesis and characterization of a polyacrylate with biphenyl pendant groups. This polymer exhibits fluorescent emission in the range of 300 to 550 nm and wide band gap. Further, the polymer is highly soluble in different organic solvents and it can be obtained by solution or bulk polymerization techniques.

## 2. Materials and methods

### 2.1. General experimental procedures

Reagents and solvents employed for the synthesis of monomer and polymers were purchased from Sigma-Aldrich Company. Acryloyl chloride was purified by distillation in presence of hydroquinone. Pyridine was distilled and dried over KOH under argon atmosphere. Tetrahydrofurane (THF) was distilled and dried with metallic sodium and benzophenone under argon atmosphere. 2,2ʹ-azobisisobutironitryle (AIBN) and benzoyl peroxide (BPO) were recrystallized in methanol. 4-biphenylmethanol was employed as received.

FT-IR spectra were acquired with a Perkin-Elmer Frontier spectrophotometer with a spectral resolution of 4 cm^−1^, samples were prepared as pellets with anhydrous KBr. Nuclear magnetic resonance (NMR) spectra were recorded with a Varian nuclear magnetic resonance 400 MHz spectrometer and it was operated at 400/100 MHz for ^1^H and ^13^C NMR respectively. Tetramethylsilane (TMS) was used as internal reference and samples were prepared with CDCl_3_ as solvent. Molecular weight of polymer was obtained by gel permeation chromatography (GPC) with an ALLIANCE 2695 WATERS chromatographer with an UV WATTERS 2998 (Photodiode Array Detector) detector and two PLgel MIXED C lineal columns. Elemental analysis (C, H, N) was carried out with a Perkin–Elmer C, H, N analyser model 2400 using acetanilide as reference. UV-Vis absorption and fluorescence spectra in solution were acquired with with a Perkin-Elmer Lambda 2S and Lambda LS55 spectrophotometers. Samples were prepared as 10 mg dm^−3^ solutions employing chloroform, *N,N*-dimethylformamide (DMF), dimethylsulfoxide (DMSO), toluene and chlorobenzene as solvents. Spectra were recorded scanning between 200 and 800 nm at room temperature. Samples were placed in quartz cuvettes with optical length of 10 mm. Optical characterization in solid state was performed with a Perkin-Elmer Lambda 35 for UV-Vis absorption and Lambda LS55 spectrophotometer for fluorescence.

The optical band gap was calculated from the UV-Vis spectrum using the Planck equation:

(1) $$E_{g}^{opt}=\frac{hc}{\lambda }$$

Where *E_g_°^pt^* corresponds to the optical band gap value between HOMO and LUMO orbitals expressed in eV; *h* is the Planck constant (4.14X10^−15^ eV∙s); and *c* represents the light velocity in the vacuum (3X10^8^ m∙s^−1^). λ is the intersection point of the tangent line to UV-Vis spectrum with x axis.

The relative quantum yield of fluorescence (*Φ*) was calculated by a comparative method using the equation

(2) $$\phi_{s}=\left(\frac{A_{x}}{A_{s}}\right)\left(\frac{F_{s}}{F_{x}}\right){\left(\frac{\eta_{s}}{\eta_{x}}\right)}^{2}\phi_{x}$$

Where *A* is the absorbance in the UV-Vis spectrum, *F* is the area under the curve of the fluorescence spectrum, *η* is the refraction index of the solvent. The *x* and *s* sub-indices appoints the standard and sample respectively. Samples were prepared as 2 mg∙dm^−3^ solutions in chloroform. Tryptophan (*Φ* = 0.14) 10^−6^ mol∙L^−1^ in water was used as standard. The Stokes shift was calculated as the difference in nm between the spectral positions of *λ_max_* of absorption and *λ_max_* of emission.

(3) $$Stokes\;shift=\;\lambda_{max}Abs-\lambda_{max}Em$$

### 2.2. Synthesis of monomer 1,1ʹ-BP4MA

**Scheme 1.**
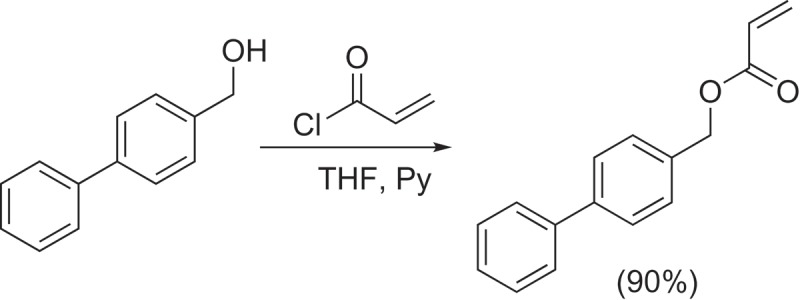
Synthesis of 1,1ʹ-BP4MA monomer.

The monomer (1,1ʹ-biphenyl)-4-ylmethyl acrylate (1,1ʹ-BP4MA) was synthetized by reaction nucleophilic acyl substitution reaction of 4-biphenylmethanol with acryloyl chloride as it is shown in the scheme 1. 1 g of 4-biphenylmethanol (5.4276 mmol) was dissolved in 30 cm^−3^ of THF freshly dried and distilled. The mixture was cooled at 5–7°C in an ice bath and then 0.45 cm^−3^ of acryloyl chloride (5.4270 mmol) and 0.45 cm^−3^ of pyridine (5.4260 mmol) were added dropwise simultaneously. The mixture was stirred for 5 h at room temperature covered of light obtaining a heterogeneous mixture containing a yellow precipitate. The precipitate was filtered, rinsed with THF and discarded. The obtained solution was distilled to remove the THF and the product was dissolved in dichloromethane and washed four times with 20 cm^−3^ of aqueous HCl solution (5% v/v). The organic phase was dried with sodium sulphate and dichloromethane was distilled to obtain the monomer spectroscopically pure as a yellow liquid. Yield: 1.164 g (90%). FT-IR (KBr) cm^−1^: 2920 νC-H; 1735 νC = O; 1640 νC = C; 1600 νC = C; 1180 νC-O. ^1^H NMR (400 MHz, CDCl_3_) δ (ppm): 7.59 (4H, m, Ar-H); 7.44 (4H, m, Ar-H); 7.35 (1H, m, Ar-H); 6.47 (1H, dd, *J* = 17.2, 0.9 Hz, CH = CH_2_); 6.18 (1H, dd, *J* = 17.2, 10.4 Hz, CH = CH_2_); 5.85 (1H, dd, *J* = 10.4, 0.9 Hz, CH = CH_2_); 5.24 (2H, s, OCH_2_). RMN-^13^C (100 MHz, CDCl_3_) δ (ppm): 165.89 (C = O); 141.07 (Ar); 140.43 (Ar); 134.62 (Ar); 131.04 (CH = CH_2_); 128.61 (Ar); 128.54 (CH = CH_2_); 128.08 (Ar); 127.26 (Ar); 127.21 (Ar); 126.93 (Ar); 65.91 (CH_2_O). Elemental analysis C_16_H_14_O_2_ (calculated) experimental: %C (80.65) 79.66; %H (5.92) 6.07.

### 2.3. Solution polymerization of 1,1ʹ-BP4MA

**Scheme 2.**
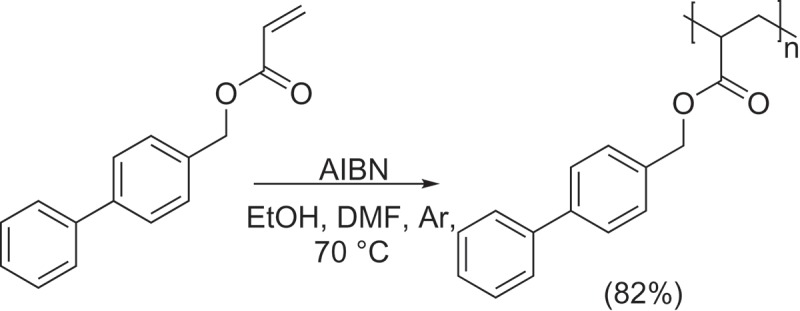
Synthesis of poly(1,1ʹ-BP4MA).

The monomer 1,1ʹ-BP4MA was polymerized via free radicals according with the Scheme 2. 1 g (4.1967 mmol) of monomer and 20 mg (2% wt.) of AIBN were dissolved in 1 mL of EtOH/DMF (80:20) in a glass test tube, then the homogeneous mixture was bubbled with argon for 20 min. Polymerization was carried out at 70°C for 15 min, using oil bath. The polymer was precipitate in acetone, filtered and dried at 60°C under vacuum for 12 h. Yellow powder. Yield: 0.8240 g (82.4%). FT-IR (KBr) cm^−1^: 2920 νC-H; 1725 νC = O; 1600 νC = C; 1170 νC-O. RMN-^1^H (400 MHz, CDCl_3_) δ (ppm): 7.29 (9H, broad signal, Ar-H); 4.94 (2H, broad signal, OCH_2_); 2.46 (1H, broad signal, CH); 1.81 (2H, broad signal, CH_2_). RMN-^13^C (100 MHz, CDCl_3_) δ (ppm): 174.12 (C = O); 139.87 (Ar); 134.14 (Ar); 128.30 (Ar); 126.38 (Ar); 65.70 (OCH_2_); 47.62 (CH); 42.53 (CH_2_). Elemental analysis C_16_H_14_O_2_ (calculated) experimental: %C (80.65) 79.66; %H (5.92) 6.07.

### 2.4. Bulk polymerization of 1,1ʹ-BP4MA

1,1-BPMA monomer was polymerized via free radicals with AIBN or BPO as thermal initiators at 70 or 90°C respectively. 60 mg of monomer and 1.2 mg of initiator (2%wt.) were placed in a test tube. The tubes were degassed by the usual freezing and thawing technique under vacuum and then sealed off. Polymerization was realized at different reaction time (15, 30 or 60 min). The percentage of insoluble polymer formed (gel content) was obtained by extracting the soluble part (sol fraction) from the cured monomer with acetone. After extraction, the gel content was dried to a constant weight. Gel content is taken as the weight percent of poly(1,1ʹ-BP4MA) obtained.

## 3. Results and discussion

### 3.1. Characterization of the 1,1ʹ-BP4MA monomer

The monomer 1,1ʹ-BP4MA is an ester obtained by reaction of 4-biphenylmethanol with acryloyl chloride. The monomer was obtained as a yellow liquid miscible with different organic solvents (toluene, CHCl_3_, AcOEt, acetone, EtOH, DMF and DMSO) and it was characterized by FTIR and NMR spectroscopies.

**Figure 1.**
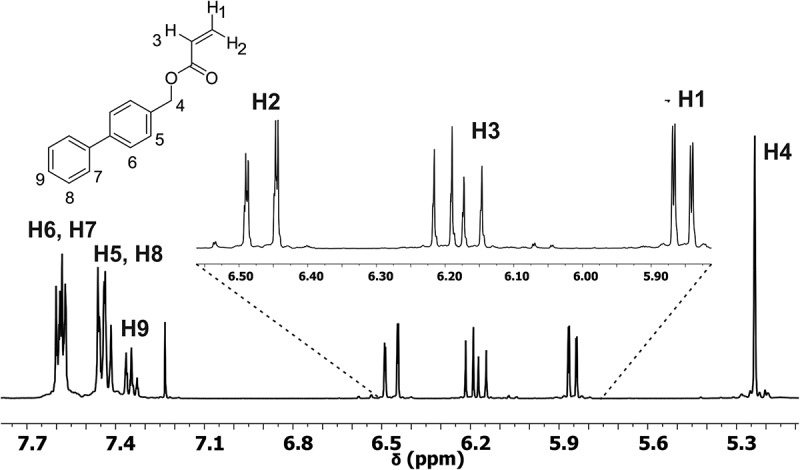
^1^H NMR spectrum of 1,1ʹ-BP4MA (400 MHz, CDCl_3_).

The most important evidence of formation of the new monomer in the FT-IR spectrum is the absorption band at 1735 cm^−1^ attributed to νC = O vibration of ester group. The presence of the double bond of alkene was confirmed by the absorption band at 1640 cm^−1^ assigned to νC = C vibration. Also, the spectrum showed the band of νC = C vibration of aromatic ring at 1600 cm^−1^. The ^1^H-NMR spectrum (Figure 1) showed three signals between 6.47 and 5.86 ppm attributed to protons of vinyl group. Signals due to aromatic protons were observed between 7.7 and 7.3 ppm. Finally, the single signal at 5.24 ppm was assigned to protons of methylene group adjacent to acrylate group. The ^13^C- NMR spectrum showed a signal at 165.89 ppm assigned to the carbon atom of carbonyl group. Signals observed at 128.54 and 131.04 ppm were assigned to the carbon atoms of vinyl group.

### 3.2. Solution polymerization of 1,1ʹ-BP4MA

**Figure 2.**
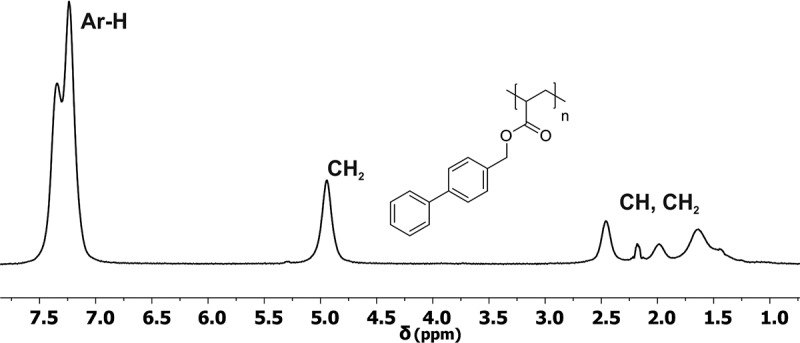
^1^H-NMR of poly(1,1ʹ-BP4MA) (400 MHz, CDCl_3_).

The homopolymer poly(1,1ʹ-BP4MA) was obtained as a yellow solid and it was characterized by FTIR and NMR. Considering that the monomer 1,1ʹ-BP4MA is a liquid compound, the first evidence of polymerization of 1,1ʹ-BP4MA was the change of the physic state obtaining poly(1,1ʹ-BP4MA) as a solid product. The FT-IR spectrum of poly(1,1ʹ-BP4MA) showed absorption bands of νC = O and νC = C vibrations of ester and aromatic ring at 1725 and 1600 cm^−1^ respectively. After polymerization, the ^1^H-NMR spectrum of poly(1,1ʹ-BP4MA) (Figure 2) did not show signals attributed to vinylic protons. The spectrum of poly(1,1ʹ-BP4MA) shows broad bands at 2.46 and 1.81 ppm, assigned to protons of polymer backbone. Aromatic protons are observed as a broad signal at 7.29 ppm and the signal due to protons of methylene was observed at 4.94 ppm. The ^13^C- NMR spectrum (Figure 4) showed two signals at 47.62 and 42.53 ppm assigned to carbon atoms of the polymer backbone. Signals observed at 128.54 and 131.04 ppm due to carbons of vinylic group of the monomer are absent in the ^13^C- NMR spectrum of polymer.

**Figure 3.**
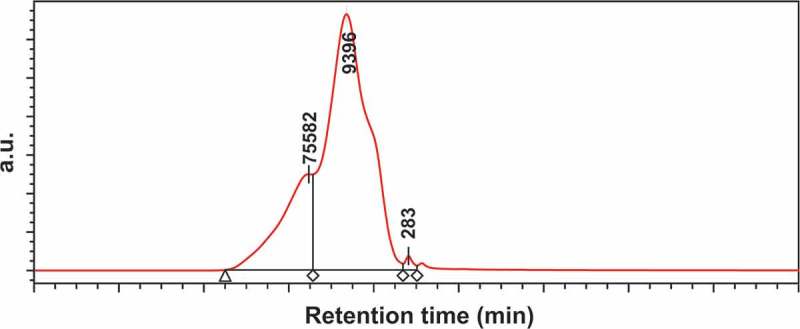
GPC chromatogram of poly(1,1ʹ-BP4MA).

**Figure 4.**
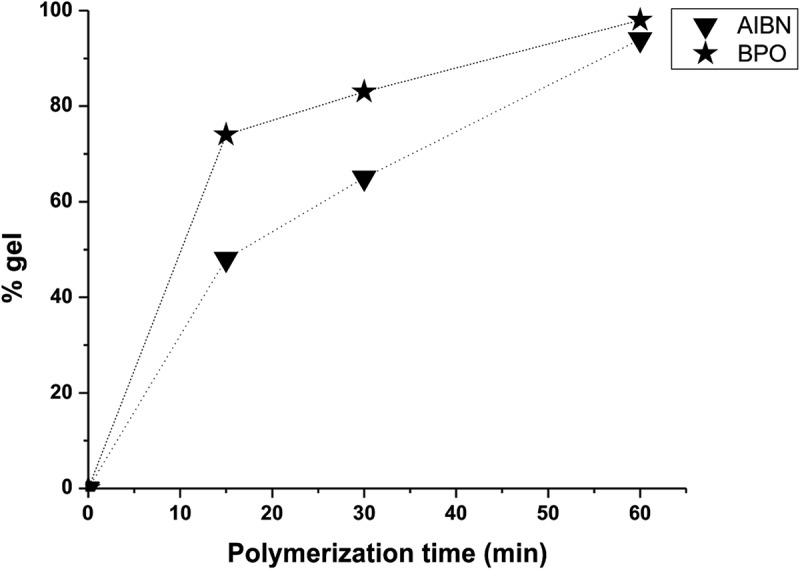
Sol-gel curves of polymerization of 1,1ʹ-BP4MA.

1,1ʹ-BP4MA is a highly reactive monomer able to polymerized to yield polymers with high molecular weight. The molecular weight of poly(1,1ʹ-BP4MA) was determinate by GPC obtaining M_n_ = 154,982 g∙mol^−1^, M_w_ = 406,063 g∙mol^−1^ and polydispersity of 2.62 (Figure 3). Poly(1,1ʹ-BP4MA) is a linear polymer with high solubility in organic solvents such CHCl_3_, toluene, benzene, DMF and DMSO, which is useful for the obtaining of films of poly(1,1ʹ-BP4MA) by different techniques.

### 3.3. Bulk polymerization of 1,1ʹ-BP4MA

Bulk polymerization of 1,1ʹ-BP4MA was realized using two different thermal initiators: AIBN and BPO at 70 and 90°C respectively with different reaction time (15, 30 or 60 min). After polymerization there was obtained a yellow and translucent solid with the mold shape. The cured polymer was treated with acetone to extract the not polymerized monomer and the gel content was calculated with the next equation

(4) $$\%\;gel=\;\left(\frac{mass\;after\;extraction}{mass\;before\;extraction}\right)*100$$

Figure 4 shows the gel percent as function of the polymerization time for the 1,1ʹ-BP4MA with both thermal initiators. AIBN and BPO leaded to obtain gel percentages close to 100% after 60 min of reaction (Figure 4). However, when polymerization is carried out with BPO, higher gel percent are obtained since the first minutes of reaction because the thermal energy is higher to promote the homolytical rupture of double bonds C = C. The high percent in gel of poly(1,1ʹ-BP4MA) are due to the high reactivity of acrylate group. From the above, 1,1ʹ-BP4MA could be applied for the fabrication of fluorescent coatings by polymerization in situ.

### 3.4. Optical properties of poly(1,1ʹ-BP4MA)

**Figure 5.**
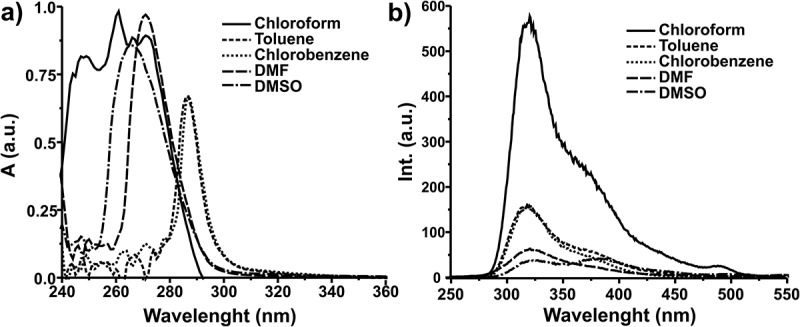
(a) UV-Vis and (b) Fluorescence spectra of poly(1,1ʹ-BP4MA) in different solvents.

Optical properties of poly(1,1ʹ-BP4MA) were studied by UV-Vis and fluorescence spectroscopies. UV-Vis spectra of poly(1,1ʹ-BP4MA) were acquired from 10 mg∙dm^−3^ solutions prepared with different solvents (chloroform, toluene, chlorobenzene, DMF and DMSO). Spectra of poly(1,1ʹ-BP4MA) acquired in different solvents are showed in the Figure 5(a) and they showed absorption bands in the UV-region. The spectrum of poly(1,1ʹ-BP4MA) acquired in chloroform showed a λ_max_ in 250 nm. The spectra acquired in other solvents showed values of λ_max_ bathochromically shifted respect to spectrum acquired in chloroform. λ_max_ was observed at 286 nm in toluene and chlorobenzene, at 269 nm in DMSO and at 271 nm in DMF respectively.

The optical band gap of poly(1,1ʹ-BP4MA) was calculated from UV-Vis spectra obtaining values between 4.11 and 4.34 eV. These results showed that poly(1,1ʹ-BP4MA) exhibit semiconductor behaviour with wide band gap, which is characteristic of fluorescent materials with emission in the UV-green region.

**Figure 6.**
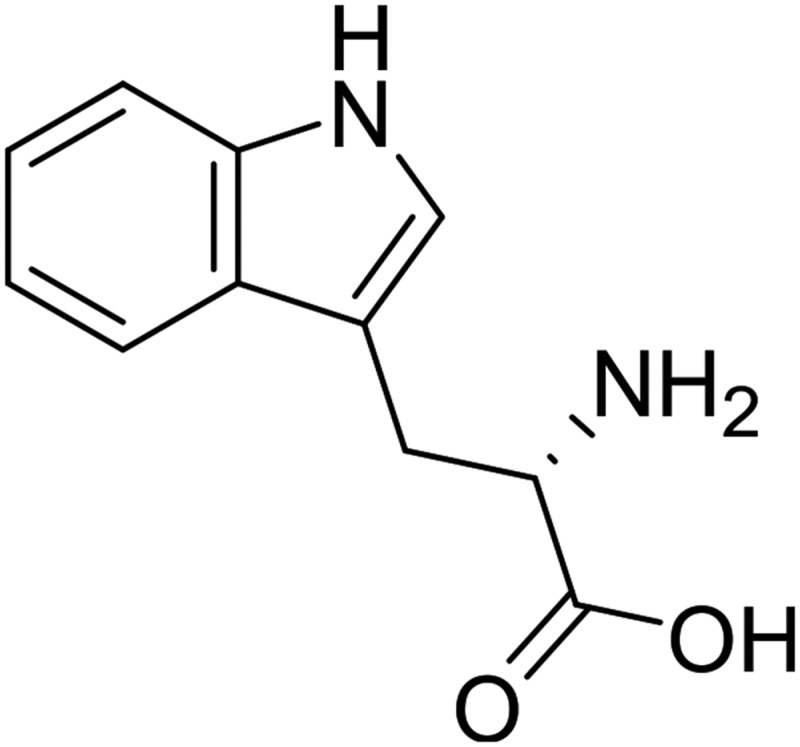
Structure of tryptophan.

Fluorescence spectra of poly(1,1ʹ-BP4MA) were acquired from 10 mg∙dm^−3^ solutions in different solvents ant they are showed in the Figure 5(b). Spectra showed wide emission bands in the range of 275–550 nm which corresponds to UV-green region. The spectrum in chloroform showed λ_max_ in 321 nm, which correspond to UV region. The spectra acquired in toluene, chlorobenzene and DMF are similar and λ_max_ was observed at 319 nm, while the spectrum obtained in DMSO two maximum points at 321 and 370 nm. The Φ of poly(1,1ʹ-BP4MA) in chloroform is 0.12 and it is comparable with the quantum yield of fluorescence of tryptophan (Figure 6). Nevertheless, the Φ of the poly(1,1ʹ-BP4MA) in toluene, chlorobenzene, DMSO and DMF was lower than Φ obtained in chloroform (Table 1), due to the low intensity of the emission band with the change of solvent. Poly(1,1ʹ-BP4MA) showed high values of Stokes Shift and they could be associated with the non-planar structure of biphenyl group which exhibit free rotation around C-C bond between aromatic rings. The results obtained from optical characterization are summarized in the Table 1.

**Table 1.** Optical properties of poly(1,1ʹ-BP4MA) in solution.

Solvent	λ_max_ absorption (nm)	E_g_°^pt^ (eV)	λ_max_ emission (nm)	Stokes shift (nm)	Φ
Toluene	286	4.21	319	33	0.07
Chlorobenzene	286	4.11	319	33	0.06
Chloroform	250	4.34	321	71	0.12
DMF	271	4.34	319	48	0.03
DMSO	269	4.21	370	101	0.03

**Figure 7.**
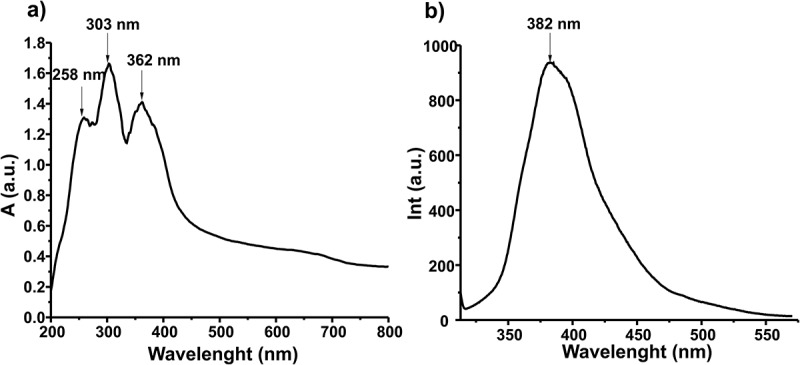
UV-Vis and fluorescence spectra of poly(1,1ʹ-BP4MA) acquired in solid state.

Finally, the optical properties of poly(1,1ʹ-BP4MA) were studied in solid state. The UV-Vis spectrum shows three wide absorption bands at 258, 303 y 362 nm. While, the fluorescence spectrum shows a wide emission band in the range of 315 y 575 nm, which corresponds to UV-green region, with λ_max_ at 382 nm. The UV-Vis and fluorescence spectra showed that the optical properties are preserved in solid state (Figure 7).

## 4. Conclusions

In summary, 1,1ʹ-BP4MA is a highly reactive monomer which could be polymerized by different techniques to obtain polymers with high molecular weight and high solubility. Fluorescent polymers can be obtained by polymerization of 1,1ʹ-BP4MA and they have emission in the UV-green region of electromagnetic spectrum. The fluorescence quantum yield of the polymer was 0.12 showing similar properties to tryptophan. The polymers obtained from 1,1ʹ-BP4MA monomer could be easily applied in the construction of fluorescent thin layers and coatings.
